# Stearoyl-CoA Desaturase 1 Activity Determines the Maintenance of DNMT1-Mediated DNA Methylation Patterns in Pancreatic β-Cells

**DOI:** 10.3390/ijms21186844

**Published:** 2020-09-18

**Authors:** Aneta M. Dobosz, Justyna Janikiewicz, Anna M. Borkowska, Anna Dziewulska, Ewelina Lipiec, Pawel Dobrzyn, Wojciech M. Kwiatek, Agnieszka Dobrzyn

**Affiliations:** 1 Laboratory of Cell Signaling and Metabolic Disorders, Nencki Institute of Experimental Biology, Polish Academy of Sciences, 02-093 Warsaw, Poland; a.dobosz@nencki.edu.pl (A.M.D.); j.janikiewicz@nencki.edu.pl (J.J.); a.dziewulska@nencki.edu.pl (A.D.); 2 Division of Interdisciplinary Research, Institute of Nuclear Physics, Polish Academy of Sciences, 31-342 Krakow, Poland; anna.maria.borkowska@uj.edu.pl (A.M.B.); Ewelina.Lipiec@ifj.edu.pl (E.L.); Wojciech.Kwiatek@ifj.edu.pl (W.M.K.); 3 Faculty of Physics, Astronomy and Applied Computer Science, Jagiellonian University, 30-348 Krakow, Poland; 4 Laboratory of Molecular Medical Biochemistry, Nencki Institute of Experimental Biology, Polish Academy of Sciences, 02-093 Warsaw, Poland; p.dobrzyn@nencki.edu.pl

**Keywords:** SCD1, DNA methylation, AMPK, epigenetic regulation, lipotoxicity

## Abstract

Metabolic stress, such as lipotoxicity, affects the DNA methylation profile in pancreatic β-cells and thus contributes to β-cell failure and the progression of type 2 diabetes (T2D). Stearoyl-CoA desaturase 1 (SCD1) is a rate-limiting enzyme that is involved in monounsaturated fatty acid synthesis, which protects pancreatic β-cells against lipotoxicity. The present study found that SCD1 is also required for the establishment and maintenance of DNA methylation patterns in β-cells. We showed that SCD1 inhibition/deficiency caused DNA hypomethylation and changed the methyl group distribution within chromosomes in β-cells. Lower levels of DNA methylation in SCD1-deficient β-cells were followed by lower levels of DNA methyltransferase 1 (DNMT1). We also found that the downregulation of SCD1 in pancreatic β-cells led to the activation of adenosine monophosphate-activated protein kinase (AMPK) and an increase in the activity of the NAD-dependent deacetylase sirtuin-1 (SIRT1). Furthermore, the physical association between DNMT1 and SIRT1 stimulated the deacetylation of DNMT1 under conditions of SCD1 inhibition/downregulation, suggesting a mechanism by which SCD1 exerts control over DNMT1. We also found that SCD1-deficient β-cells that were treated with compound c, an inhibitor of AMPK, were characterized by higher levels of both global DNA methylation and DNMT1 protein expression compared with untreated cells. Therefore, we found that activation of the AMPK/SIRT1 signaling pathway mediates the effect of SCD1 inhibition/deficiency on DNA methylation status in pancreatic β-cells. Altogether, these findings suggest that SCD1 is a gatekeeper that protects β-cells against the lipid-derived loss of DNA methylation and provide mechanistic insights into the mechanism by which SCD1 regulates DNA methylation patterns in β-cells and T2D-relevant tissues.

## 1. Introduction

The emergence of type 2 diabetes (T2D) as a global pandemic is a major issue. The two central abnormalities that characterize the pathophysiology of T2D are insulin resistance in peripheral tissues and the progressive loss of β-cell function in pancreatic islets [1,2,3]. Accumulating evidence indicates that epigenetic modifications, genetic background, environmental factors, and the dysregulation of lipid homeostasis are primary reasons underlying β-cell dysfunction and T2D development [2]. 

DNA methylation and other epigenetic alterations, such as histone methylation and acetylation, regulate gene expression and are crucial for maintaining genomic integrity and chromosome structure. DNA methylation is a chemical post-replicative modification whereby a methyl group (-CH_3_) is covalently added to the fifth carbon of the cytosine ring of 5′-to-3′-oriented CG dinucleotides, which are known as CpG sites [4]. Regions with a high frequency of CpG sites are often found in gene promoters. Cytosine methylation within the promoter region is known to inversely correlate with expression of the gene by affecting the binding of transcription factors and methylcytosine-binding proteins to the DNA and heterochromatin formation [5]. In mammals, DNA methylation patterns are generated and maintained by a family of DNA methyltransferases (DNMTs). This process is highly dependent on the availability of S-adenosyl methionine (SAM), a primary methyl group donor [6]. DNMT1 is the most abundant DNA methyltransferase in adult somatic cells and regarded as a maintenance methyltransferase. DNMT1 preferentially catalyzes the addition of the -CH_3_ group to the hemi-methylated DNA strand during the S-phase of replication [7]. However, the significant capacity of DNMT1 for de novo methylation was also reported [8]. DNMT1 activity and protein stability are highly regulated by various post-translational modifications, such as acetylation, methylation, and ubiquitination—through interactions with other epigenetic effectors, such as the NAD-dependent deacetylase sirtuin-1 (SIRT1) [9,10].

The establishment and preservation of DNA methylation patterns along the genome are critically important for both pancreatic organogenesis and mature β-cell function and survival [11,12]. A whole-genome bisulfite sequencing study identified 25,820 differentially methylated regions (DMRs) and 457 genes that presented both DMRs and significant changes in expression in pancreatic islets from T2D donors, including loci with a key function in islet biology (e.g., *PDX1*, *ADCY5*, and *SLC2A2*) [13]. DNMT1-deficient pancreatic β-cells were deprived of their epigenetic constraints and converted into α-cells through hypomethylation of the *ARX* promoter region [11]. Evidence shows that changes in DNA methylation are also part of the functional adaptation of β-cells to lipotoxicity that is related to T2D pathogenesis [14]. Pancreatic islets that were exposed to palmitic acid (16:0) exhibited dysregulation of DNA methylation and changes in expression of genes that are involved in insulin signaling, lipid metabolism, and energy homeostasis [14].

Stearoyl-CoA desaturase 1 (SCD1) is a pivotal enzyme that is involved in palmitic acid metabolism and has a protective action against lipotoxicity in β-cells [15,16]. SCD1 catalyzes insertion of the double bond at the delta-9 position of 12–19 carbon in saturated fatty acids (SFAs; preferentially palmitate (16:0) and stearate (18:0)), thereby converting them to monounsaturated fatty acids (MUFAs; palmitoleate (16:1n-7) and oleate (18:1n-9)) [16]. Long-chain SFAs are reportedly more cytotoxic than long-chain MUFAs. SCD1 activity has crucial physiological importance for proper β-cell function, and its ablation is associated with β-cell failure and T2D development [15,17,18]. SCD1 deficiency results in lower glucose-stimulated insulin secretion, proliferation, and apoptosis induction in β-cells [19]. Furthermore, the inhibition of SCD1 activity affects autophagosome-lysosome fusion through perturbations in cellular membrane integrity, leading to an aberrant stress response and β-cell failure [19]. Our previous studies demonstrated the well-established role of SCD1 in the control of intracellular SFA/MUFA equilibrium and showed that SCD1 is also involved in epigenetic regulation [20,21]. The inhibition of SCD1 was shown to alter DNA methylation levels in 3T3-L1 adipocytes [21]. Additionally, SCD1 expression affects the global acetylation and methylation levels of lysine 9 on histone H3 through an increase in the activity of adenosine monophosphate-activated protein kinase (AMPK) and SIRT1 in skeletal muscle [20]. However, to our knowledge, no study has evaluated the orchestration of epigenetic events by SCD1 in pancreatic islets.

Considering the possible interplay between SCD1 and epigenetic regulation, we investigated the role of SCD1 in maintaining the DNA methylation profile in pancreatic β-cells. We found that SCD1 inhibition/gene silencing altered the distribution of -CH_3_ groups within chromosomes and elicited DNA hypomethylation in β-cells. These effects appeared to be mediated by the AMPK/SIRT1-dependent downregulation of DNMT1.

## 2. Results

### 2.1. SCD1 Deficiency Is Associated with a Decrease in gDNA Methylation Levels in Pancreatic β-Cells

**Figure 1:**
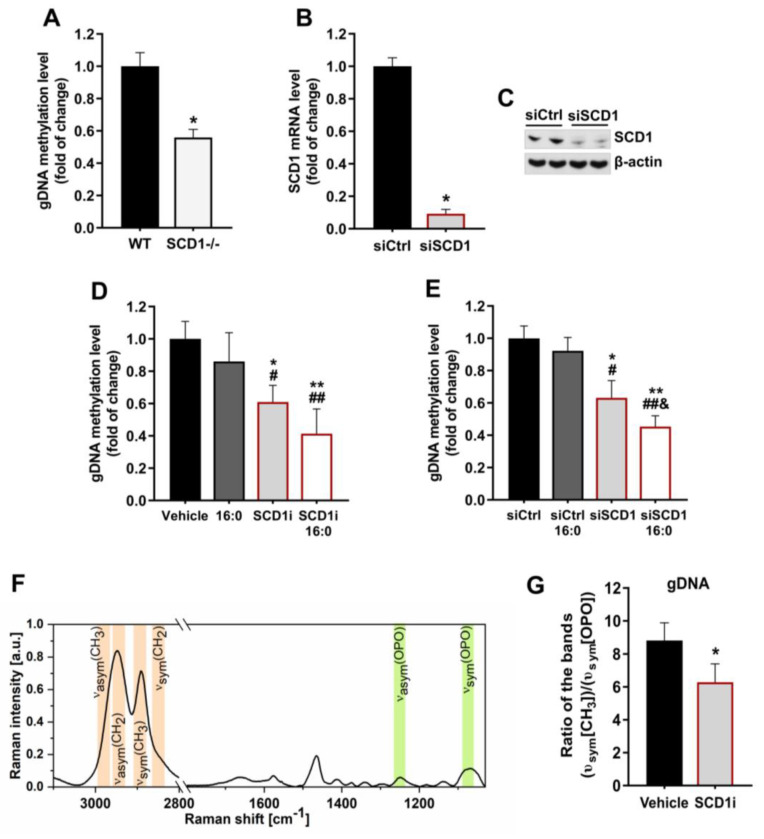
Stearoyl-CoA desaturase 1 (SCD1) downregulation leads to global DNA (gDNA) hypomethylation in pancreatic islets and INS-1E cells. (**A**) gDNA methylation level in pancreatic islets that were isolated from wildtype (WT) and SCD1^−/−^ mice. *n* = 6. * *p* < 0.05, vs. WT. (**B**,**C**) Measurement of SCD1 silencing (siSCD1) efficiency by analyzing SCD1 mRNA levels by real-time PCR (**B**) and Western blot (**C**). (**D**,**E**) gDNA methylation level in INS-1E cells that were treated with the SCD1 inhibitor A939572 (SCD1i) (**D**) or an siRNA against SCD1 (siSCD1) (**E**) and palmitic acid (16:0). gDNA methylation levels were evaluated by HpaII/MspI digestion. (**F**) Average Raman spectrum of DNA that was isolated from INS-1E cells (average of 40 spectra). (**G**) The estimation of gDNA methylation levels, based on Raman spectroscopic analysis, in control and A939572-treated INS-1E cells is presented as an average integration value (*n* = 10) of the band in the spectral range of 2920–2870 cm^−1^ (υ_sym_(CH_3_)), corresponding to the distribution of -CH_3_ groups and normalized to the band in the spectral range of 1110–1030 cm^−1^ (υ_sym_(OPO)), corresponding to the distribution of DNA. The data are expressed as mean ± SD. * *p* < 0.05, ** *p* < 0.005, vs. vehicle/siCtrl; # *p* < 0.05, ## *p* < 0.005 vs. 16:0/16:0 siCtrl; & *p* < 0.05, vs. siSCD1. The data are expressed as the mean ± SD of three independent experiments.

The level of global DNA (gDNA) methylation in pancreatic islets that were isolated from SCD1^−/−^ mice significantly decreased by 40% compared with WT islets (Figure 1A). To investigate the effect of SCD1 on the regulation of DNA methylation patterns in pancreatic INS-1E cells, we transfected INS-1E cells with specific siRNA, which decreased SCD1 mRNA levels by ~90% (Figure 1B) and SCD1 protein levels by >60% (Figure 1C). We also decreased SCD1 activity in INS-1E cells using the selective pharmacological inhibitor A939572. The cells were incubated with palmitate (16:0), which has been shown to augment the severity of the SCD1 deficiency-related stress response in insulin-secreting MIN6 cells [22]. Both SCD1 inhibition and the genetic ablation of SCD1 by siRNA decreased gDNA methylation levels by ~40% compared with control cells (Figure 1D,E). The treatment of INS-1E cells with palmitate itself did not significantly affect gDNA methylation levels. However, gDNA methylation levels in SCD1-deficient INS-1E cells that were incubated with palmitate decreased by nearly 60% compared with controls and were higher than in cells that were treated with the SCD1 inhibitor, or siRNA against SCD1 alone (Figure 1D,E).

We also evaluated the effect of SCD1 inhibition on gDNA methylation levels based on spectroscopic Raman analysis. We first analyzed the average Raman spectrum of DNA that was isolated from INS-1E cells. The Raman spectrum contained bands that were characteristic of the stretching of bonds between phosphate and oxygen atoms in the DNA backbone at 1250 cm^−1^ (υ_asym_(OPO)) and 1090 cm^−1^ (υ_sym_(OPO)) and bands that were characteristic of -CH_3_ group vibrations at 2975 cm^−1^ (υ_asym_(CH_3_)) and 2890 cm^−1^ (υ_sym_(CH_3_); Figure 1F). We then integrated the band in the spectral range of 2920–2870 cm^−1^, which reflected the quantitative amount of -CH_3_ groups in DNA from control INS-1E cells, and cells that were treated with the SCD1 inhibitor. Consistent with the enzymatic results, the inhibition of SCD1 activity led to the loss of -CH_3_ groups in DNA and a reduction in gDNA methylation levels in INS-1E cells compared with controls (Figure 1G).

### 2.2. Inhibition of SCD1 Activity Leads to Changes in Methyl Group Distribution within Chromosomes in Pancreatic β-Cells

**Figure 2:**
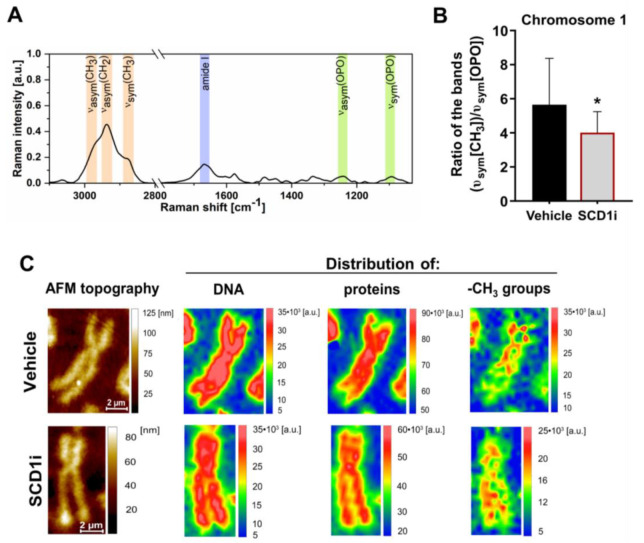
Raman spectroscopic analysis of the effect of SCD1 inhibition on the spatial distribution of -CH_3_ groups within single chromosome 1 in INS-1E cells. (**A**) Average Raman spectrum of chromosomes 1 (*n* = 15, spectra obtained using empty modeling analysis) that were isolated from INS-1E cells (smoothed, Savitzky–Golay filter, second order, 11 pt). (**B**) Estimation of global methylation levels in single chromosome 1 from control INS-1E cells and cells that were treated with the SCD1 inhibitor, presented as an average integration value (*n* = 6) of the band in the spectral range of 2900–2850 cm^−1^ (υ_sym_(CH_3_)), corresponding to the distribution of -CH_3_ groups and normalized to the band in the spectral range of 1110–1070 cm^−1^ (υ_sym_(OPO)), corresponding to the distribution of DNA. (**C**) Atomic force microscopy (AFM) and Raman spectroscopic maps of chromosome 1—isolated from INS-1E cells, showing the topography of single chromosome 1 and the spatial distribution of integrated bands, corresponding to the distribution of DNA, proteins (mainly histones), and methyl groups. The data are expressed as mean ± SD. * *p* < 0.01, vs. vehicle.

To further assess the role of SCD1 in the control of DNA methylation, we estimated the effect of SCD1 inhibition on -CH_3_ group distribution within single chromosome 1 from INS-1E cells. Using Empty modeling analysis, we obtained average Raman spectra of single chromosome 1 that were collected from control and SCD1 inhibitor-treated INS-1E cells (Figure 2A). Based on the integrity of the band in the spectral range of 2900–2850 cm^−1^, we estimated that SCD1 inhibition decreased methylation levels in chromosome 1 in INS-1E cells by ~26% compared with control cells (Figure 2B).

To characterize the morphology of metaphase chromosomes, we performed atomic force microscopy (AFM) topographic imaging. We analyzed the spatial distribution of the integrated band characteristic of the stretching of bonds between phosphate and oxygen atoms in the DNA backbone in the spectral range of 1280–1215 cm^−1^ (υ_asym_(OPO)), which refers to the DNA distribution, and the amide I band in the spectral range of 1695–1630 cm^−1^, that conforms to the distribution of proteins (mainly histones). We also investigated the spatial distribution of the integrated band in the spectral range of 2900–2850 cm^−1^ (υ_sym_(CH_3_)) that corresponded to the arrangement of -CH_3_ groups [23,24]. The distribution of proteins and DNA within the chromosomes was similar in the SCD1 inhibitor-treated and control samples (Figure 2C). Interestingly, we found that the inhibition of SCD1 activity in INS-1E cells affected the spatial spreading and location of -CH_3_ groups over an area of chromosome 1. The main difference between the distribution of -CH_3_ groups within chromosome 1—that was isolated from control INS-1E cells and INS-1E cells that were treated with the SCD1 inhibitor—was related to homogeneity (Figure 2C). In the case of chromosome 1 from control cells, the -CH_3_ group distribution was heterogeneous, and the level of methylation was the highest in the region of the p arm and centromere of the chromosome. The -CH_3_ group distribution was more homogeneous within chromosome 1 from INS-1E cells that were treated with the SCD1 inhibitor compared with controls (Figure 2C).

### 2.3. Ablation of SCD1 Activity and Expression Decreases DNMT1 Protein Levels in Pancreatic β-Cells

**Figure 3:**
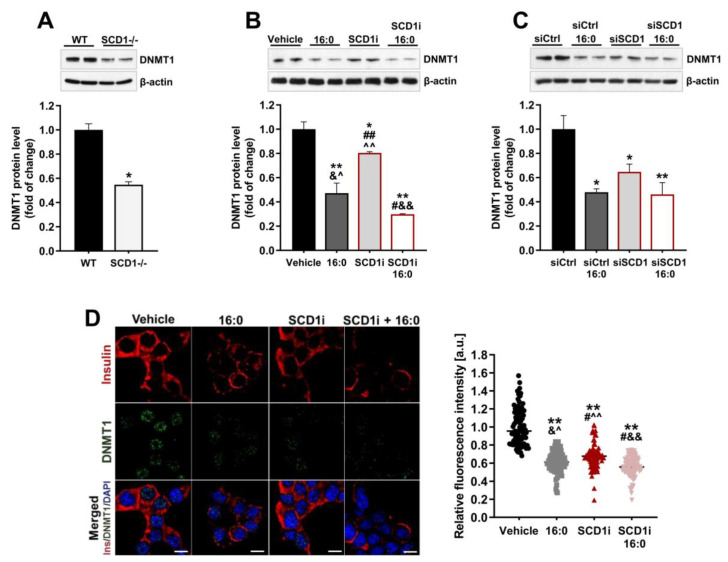
DNA methyltransferase 1 (DNMT1) is downregulated in SCD1-deficient pancreatic islets and INS-1E cells. (**A**) DNMT1 protein levels in SCD1^−/−^ pancreatic islets and WT islets. *n* = 6. * *p* < 0.05, vs. WT. (**B**,**C**) Western blot analysis of DNMT1 protein levels in INS-1E cells after SCD1 inhibition with A939572 (SCD1i) (**B**) or silencing of SCD1 gene expression by siRNA (siSCD1) (**C**) and palmitate (16:0) treatment. (**D**) Immunofluorescence staining of DNMT1 and quantitative analysis of DNMT1 content in nuclei in INS-1E cells that were treated with the SCD1 inhibitor. The data are expressed as mean ± SD. Scale bar = 10 μm. * *p* < 0.05, ** *p* < 0.005, vs. vehicle/siCtrl; # *p* < 0.05, ## *p* < 0.005, vs. 16:0; & *p* < 0.05, && *p* < 0.005, vs. siSCD1i; ^ *p* < 0.05, ^^ *p* < 0.005, vs. SCD1i+16:0.

To further investigate the role of SCD1 in the preservation of DNA methylation patterns in β-cells, we evaluated the impact of SCD1 deficiency on DNMT1, which is required for maintaining the DNA methylation profile [5]. We found that SCD1 knockdown reduced the level of DNMT1 protein by nearly 50% in pancreatic islets compared with WT islets (Figure 3A). Both the impairment of SCD1 activity and downregulation of SCD1 gene expression in INS-1E cells decreased DNMT1 protein levels by approximately 20% and 35%, respectively (Figure 3B,C). INS-1E cells that were incubated with palmitate were characterized by a ~55% reduction in DNMT1 protein levels. In groups of cells that were treated in parallel with the SCD1 inhibitor or siRNA against SCD1 and palmitate, the protein content of DNMT1 decreased by 60–70% compared with control cells (Figure 3B,C).

To support the immunoblot results, we detected DNMT1 in INS-1E cells with the inhibition of SCD1 activity by immunofluorescence staining. The visualization and quantification of fluorescently labeled DNMT1 indicated that SCD1 inhibition decreased DNMT1-positive staining in cell nuclei, which was consistent with protein content (Figure 3D). Treatment with palmitate enhanced the lowering effect of SCD1 downregulation on the fluorescence intensity of DNMT1 (Figure 3D). Additionally, consistent with previous reports [19,22], we observed a significant reduction in insulin-positive staining in SCD1-inhibited and palmitate-treated pancreatic β-cells (Figure 3D).

### 2.4. Reduction in SCD1 Activity/Expression Leads to the Activation of AMPK and Increases SIRT1 Protein Expression in Pancreatic β-Cells

**Figure 4:**
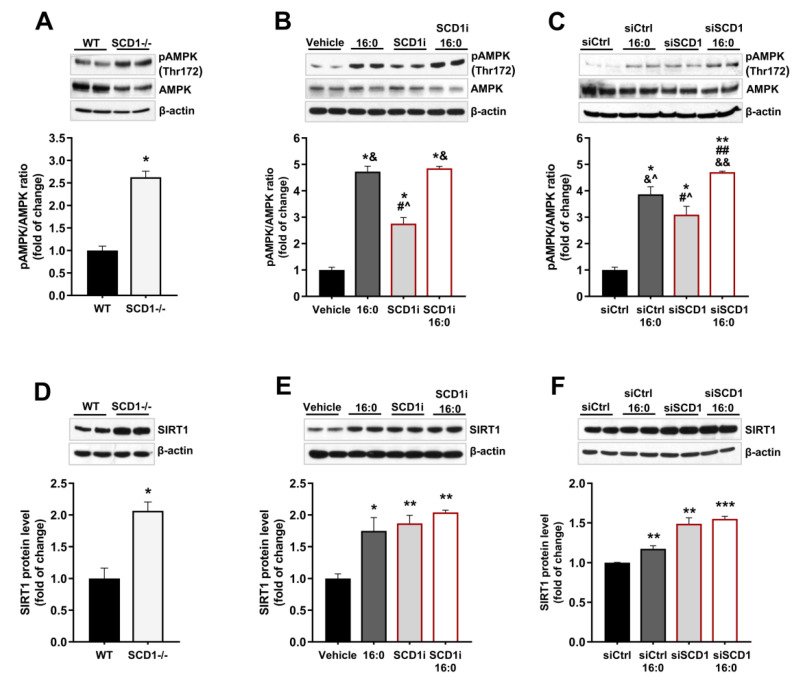
SCD1 regulates adenosine monophosphate-activated protein kinase (AMPK) and NAD-dependent deacetylase sirtuin-1 (SIRT1) in pancreatic islets and INS-1E cells. (**A**,**D**) Western blot analysis of AMPK phosphorylation at Thr172 (**A**) and the level of SIRT1 protein (**D**) in pancreatic islets from WT and SCD1^−/−^ mice. *n* = 6. * *p* < 0.05, vs. WT. (**B**,**C**,**E**,**F**) AMPK phosphorylation at Thr172 and SIRT1 protein levels in INS-1E cells that were treated with the SCD1 inhibitor A939572 (**B**,**E**) or siRNA against SCD1 (**C**,**F**) and palmitic acid (16:0). The data are expressed as mean ± SD of three independent experiments. * *p* < 0.05, ** *p* < 0.01, *** *p* < 0.001, vs. vehicle/siCtrl; # *p* < 0.05, ## *p* < 0.005 vs. 16:0/16:0 siCtrl; & *p* < 0.05, && *p* < 0.005 vs. SCD1i/siSCD1; ^ *p* < 0.05, vs. SCD1i/siSCD1+16:0.

To investigate the influence of SCD1 deficiency on the AMPK/SIRT1 axis in pancreatic islets in WT and SCD1^−/−^ mice, we measured SIRT1 protein levels and the phosphorylation of AMPK at Thr172, which is crucial for its activation [25]. Both AMPK phosphorylation and SIRT1 protein levels were elevated more than two-fold in SCD1^−/−^ pancreatic islets compared with WT islets (Figure 4A,D).

Almost a three-fold increase in AMPK phosphorylation was also observed in INS-1E cells that were treated with the SCD1 inhibitor (Figure 4B) and siRNA against SCD1 (Figure 4C) compared with control cells. In cells that were treated with palmitate, AMPK phosphorylation at Thr172 was an average of more than four-fold higher compared with untreated cells. In SCD1-deficient INS-1E cells that were incubated with palmitate, the extent of AMPK phosphorylation increased nearly two-fold compared with cells that were treated only with the SCD1 inhibitor or siRNA against SCD1 (Figure 4B,C). Furthermore, the inhibition of SCD1 activity and downregulation of SCD1 gene expression in INS-1E cells increased SIRT1 protein levels by approximately 80% and 50%, respectively (Figure 4E,F). An average 45% increase in SIRT1 protein content was observed in INS-1E cells that were stimulated with palmitate. However, no significant difference in SIRT1 protein levels was found between SCD1-deficient INS-1E cells and SCD1-deficient INS-1E cells that were treated with palmitate (Figure 4E,F).

### 2.5. SCD1 Inhibition/Downregulation Is Related to an Increase in the Deacetylation of DNMT1 by SIRT1

**Figure 5:**
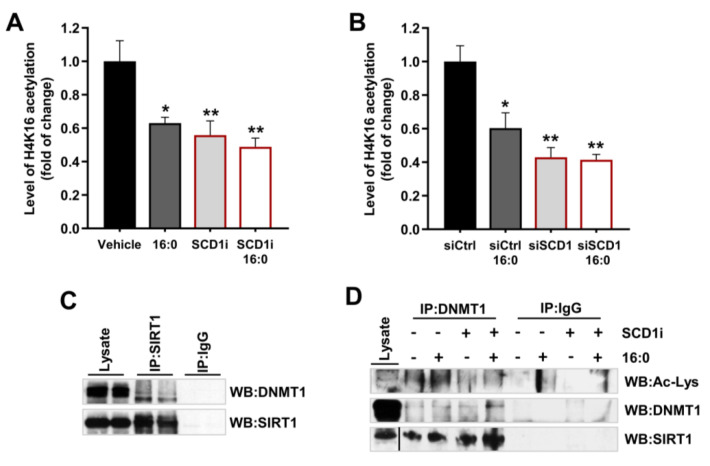
Downregulation of SCD1 elevates SIRT1 activity in INS-1E cells and promotes the deacetylation of DNMT1 by SIRT1. (**A**,**B**) Global acetyl histone H4K16 levels in INS-1E cells (indirect indicator of SIRT1 deacetylase activity) after the pharmacological inhibition of SCD1 (SCD1i) (**A**) or silencing of SCD1 gene expression (**B**) and palmitate (16:0) treatment. The data are expressed as the mean ± SD of three independent experiments. * *p* < 0.05, ** *p* < 0.005, vs. vehicle/siCtrl. (**C**) Mock precipitates (IgG control) and anti-SIRT1 immunoprecipitates from whole INS-1E cell lysates that were analyzed by Western blot using an antibody against DNMT1. (**D**) Control and anti-DNMT1 immunoprecipitates from INS-1E cells that were treated with the SCD1 inhibitor A929371 (SCD1i) and palmitate (16:0) and immunoblotted with SIRT1 and anti-acetylated lysine (Ac-Lys) antibody.

To investigate whether the higher levels of SIRT1 protein in SCD1-deficient INS-1E cells are followed by higher deacetylase activity, we measured the level of histone H4 acetylation at lysine 16 (H4K16), which is directly associated with and controlled by SIRT1 [26,27]. In INS-1E cells that were treated with the SCD1 inhibitor, H4K16 acetylation levels decreased by over 40% (Figure 5A), whereas the downregulation of SCD1 gene expression decreased H4K16 acetylation levels by nearly 60% (Figure 5B). Treatment with palmitate itself reduced the level of H4K16 acetylation by approximately 40%. Similar to SIRT1 protein levels, no significant difference in the level of H4K16 acetylation was observed between INS-1E cells that were treated with the SCD1 inhibitor or siRNA against SCD1 and SCD1-deficient INS-1E cells subjected to palmitate treatment (Figure 5A,B).

To investigate whether DNMT1 is a substrate of SIRT1 in INS-1E cells, we performed co-immunoprecipitation of these two proteins. The analysis showed that the antibody to endogenous SIRT1 coprecipitated endogenous DNMT1 (Figure 5C) and vice versa (Figure 5D). In control samples that did not contain specific antibodies against SIRT1 or DNMT1, the precipitation of neither DNMT1 (Figure 5C) nor SIRT1 (Figure 5D) was detected. We also measured the level of DNMT1 acetylation by blotting mock precipitates (IgG control) and anti-DNMT1 precipitates with anti-acetyl lysine antibody. We found that DNMT1 underwent hypoacetylation in SCD1-inhibited INS-1E cells (Figure 5D). In samples that were incubated with palmitate itself, the level of DNMT1 acetylation was greater than in untreated cells, but the application of palmitate did not elevate DNMT1 acetylation in SCD1-deficient INS-1E cells. In both precipitates of INS-1E cells that were treated with the SCD1 inhibitor and palmitate, we also detected a higher level of SIRT1 protein than in samples that were not incubated with the SCD1 inhibitor and palmitate (Figure 5D).

### 2.6. Inhibition of AMPK Upregulates DNMT1 in β-Cells and Partially Restores the Level of gDNA Methylation under Conditions of SCD1 Depletion

**Figure 6:**
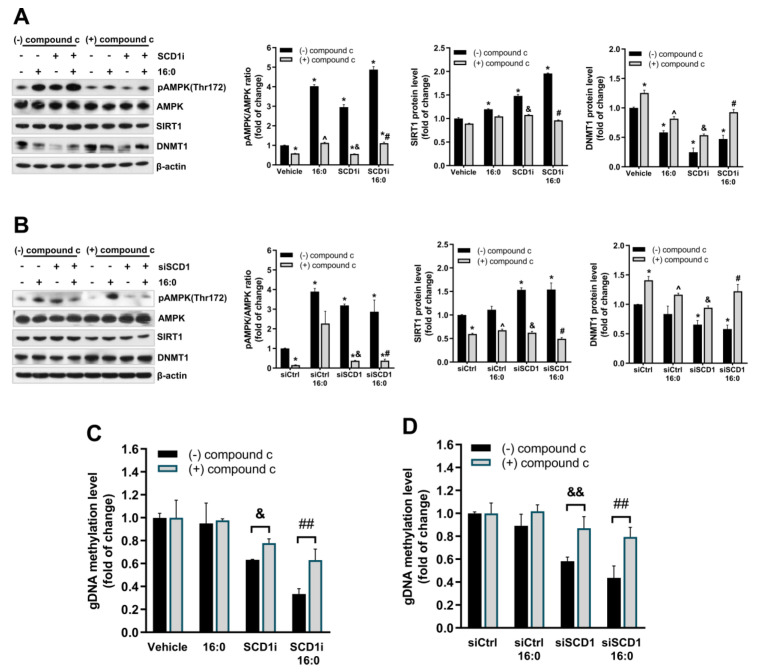
Inhibition of AMPK in SCD1-deficient INS-1E cells increases DNMT1 protein levels and prevents gDNA hypomethylation under conditions of SCD1 deficiency. (**A**,**B**) DNMT1 levels in INS-1E cells that were co-treated with the SCD1 inhibitor A939572 (SCD1i) (**A**), siSCD1 (**B**), palmitic acid (16:0), and 10 µM compound c to block AMPK. The levels of AMPK phosphorylation at Thr172 (pAMPK Thr172) and SIRT1 and DNMT1 protein content were measured by Western blot. (**C**,**D**) Effect of AMPK inhibition by compound c on gDNA methylation levels in INS-1E cells with the inhibition of SCD1 activity (**C**) and SCD1 gene silencing (siSCD1) (**D**). * *p* < 0.05, vs. Vehicle/siCtrl; ^ *p* < 0.05, vs. 16:0/16:0+siCtrl, & *p* < 0.05, && *p* < 0.005, vs. siSCD1/SCD1i; # *p <* 0.05, ## *p* < 0.005, vs. SCD1i/siSCD1+16:0. The data are expressed as the mean ± SD of three independent experiments. AMPK: adenosine monophosphate-activated protein kinase; SIRT1: NAD-dependent deacetylase sirtuin-1.

To test the hypothesis that alterations of the induction of DNMT1-dependent gDNA methylation that is caused by SCD1 inhibition rely on AMPK/SITR1 axis stimulation in β-cells, we pharmacologically blocked AMPK activity with compound c. The inhibition of AMPK by compound c was accompanied by a decrease in SIRT1 protein levels in SCD1-deficient and palmitate-treated cells (Figure 6A,B).

Furthermore, the inhibition of AMPK substantially increased DNMT1 protein levels in INS-1E cells that were incubated with palmitate and the SCD1 inhibitor (Figure 6A) or the siRNA against SCD1 (Figure 6B) compared with cells that were not treated with compound c. Interestingly, the inhibition of AMPK by compound c protected against gDNA hypomethylation in SCD1-deficient INS-1E cells (Figure 6C,D). Moreover, the effect of AMPK inhibition on the level of gDNA methylation under conditions of SCD1 deficiency was more pronounced in cells that were treated with palmitate (Figure 6C,D). In groups of cells that were treated with both the SCD1 inhibitor or siRNA against SCD1 and compound c, the decrease in gDNA methylation levels was ~25% lower than in cells that were incubated only with the SCD1 inhibitor or siRNA (Figure 6C,D). Additionally, the application of compound c increased the level of gDNA methylation by ~40% in SCD1-deficient INS-1E cells that were stimulated with palmitate, compared with the group of SCD1-deficient INS-1E cells incubated with palmitate but untreated with compound c (Figure 6C,D).

## 3. Discussion

Epigenetic alterations, such as DNA methylation, link adaptive gene expression profiles to environmental stimuli during the progression of diabetes [2,3]. Hyperlipidemia is a primary metabolic stressor that is associated with T2D. β-cells are extremely susceptible to high lipid levels and subsequent lipotoxicity. Several studies have reported that the methylation status of key loci of β-cell function (e.g., the *INS* gene that encodes insulin [28]), can be altered in the T2D in response to lipotoxicity [13,14,29,30]. Elucidation of the molecular mechanisms that mediate the interaction between environmental exposure and gene expression and the preservation of β-cell function under lipotoxic conditions is a key priority for T2D management. In the present study, we found that SCD1, a lipogenic enzyme that protects β-cells against lipotoxicity [15,16], is an essential component of the epigenetic regulatory network in β-cells. We found that SCD1 activity is required for maintaining DNA methylation patterns and chromosomes in pancreatic β-cells. We also revealed a mechanism by which SCD1 may control gDNA methylation, which is based on the dependence of DNMT1 regulation on activation of the energy sensors AMPK and SIRT1.

The lower activity and expression of SCD1 are related to an oversupply of SFAs to MUFAs and thus lipotoxicity, which contributes to β-cell failure and T2D progression [19,31]. The fatty acid-induced effect on β-cells depends on both level of fatty acid desaturation and time of exposure hence SCD1 is a main brake on palmitate toxicity in β-cells [32,33]. The overexpression of SCD1 prevents palmitate-induced endoplasmic reticulum stress and apoptosis in several cell types, including MIN6 β-cells and human pancreatic islets [32,34,35]. In addition, activation of liver X receptor (LXR), retinoid X receptor (RXR) or peroxisome proliferator-activated receptor α (PPARα) in β-cells induced the expression of SCD1, which has also been shown to protect against palmitate-induced toxicity [36]. In contrast, inhibition of SCD1 activity negatively affected the autophagy outcome and ER stress response, and led to the β-cell demise [19]. Mice lacking SCD1 in the BTBR leptin ob/ob background exhibited decline in a glucose-stimulated insulin secretion and a subpool of β-cells displayed hallmarks of SFA-induced lipotoxicity [15]. Furthermore, there was a significant increase in the expression level of SCD1 transcript in islets of prediabetic 6 week old ZDF rats in comparison to islets of 12 week old rodents with onset of overt diabetes [34]. Noteworthy, palmitate enhances some of the effects of SCD1 deficiency in β-cells [22,37]. Furthermore, since oleate can overcome some of the effects of SCD1 deficiency in hepatocytes [38], and given the important role of oleate in regulation of β-cell function [39], it is possible that the effects of SCD1 deficiency in β-cells may be mediated by oleate. However, more studies are needed to address this issue.

In the present study, we found that SCD1 downregulation/inhibition resulted in the global hypomethylation of DNA in pancreatic islets and the INS-1E β-cell line and changes in spatial distribution of methyl groups within single chromosome 1. Observations of global DNA methylation levels were made using classical electrophoresis and DNA cleavage by restriction enzymes, and they were complemented by Raman spectroscopy. Vibrational spectroscopy is a novel tool for DNA methylation studies and it was chosen by us because of its ability to analyze the molecular and chemical structure of DNA and chromosomes. Raman spectroscopy is a label-free, non-invasive and marker-independent method [40,41]. The undeniable advantage of Raman spectroscopy is the ability to obtain complete chemical information in one spectrum, which enables simultaneous analysis of the chemical structure of proteins, DNA, lipids, carbohydrates, and other molecules in the studied sample [40]. What is more, it gives access to information on the secondary structure of proteins or DNA conformation based on the position of characteristic Raman bands. When combined with microscopy, it also enables one to follow the distribution of chosen chemical components in single cells or chromosomes with the submicron spatial resolution. However, there are some limitations. Raman spectroscopy is not yet a well-established method for DNA methylation analysis [40]. Observed Raman bands of such complicated samples as chromosomes, cells or tissues are a sum of contributions from vibrations of various molecules at different conformations, which makes analysis advanced, and usually, a combination of Raman spectroscopy and multivariate statistical analysis is required to interpret the obtained results [40,41]. What is more, the pre-processing of spectra and different approaches applied in the analysis could have an impact on the final results. Some non-linear effects in Raman spectroscopy, such as resonances in hemoproteins and self-absorption of samples, could influence band intensities and yet influence the quantitative analysis of samples [42].

The administration of palmitate under conditions of SCD1 deficiency enhanced the lowering effect of SCD1 ablation on gDNA methylation, supporting previous studies that reported that the effects of palmitate toxicity were augmented by SCD1 depletion in β-cells [22,34]. The treatment of INS-1E cells with palmitate itself slightly, but non-significantly, decreased gDNA methylation levels under the present experimental conditions. The impact of palmitate on genome-wide mRNA expression and DNA methylation patterns in human pancreatic islets was studied in detail by Hall et al. Similar to the present results, Hall et al. showed that the exposure of human pancreatic islets to palmitate has only minor effects on gDNA methylation levels in pancreatic islets [14]. Interestingly, a high proportion of hypomethylation of CpG sites, especially CpGs in regulatory sequences of the genes, relative to hypermethylation events was observed in diabetic islets [29,30]. These findings suggest that some of the changes in methylation that were observed in SCD1-deficient islets and INS-1E cells, may overlap with methylation aberrations that are characteristic of T2D.

In our previous study, we found that SCD1 was a potent regulator of adipocyte and white adipose tissue inflammation that governs the expression of inflammatory cytokines via changes in promoter methylation [21]. Interestingly, in 3T3-L1 adipocytes, SCD1 inhibition/gene silencing resulted in gDNA hypermethylation, and this phenomenon was not associated with expression of the DNA methyltransferases DNMT1, DNMT3a, or DNMT3b [21]. In contrast, in pancreatic islets and INS-1E cells, the decrease in gDNA methylation that was related to SCD1 deficiency corresponded to lower DNMT1 protein levels. DNMT1, in addition to its methyltransferase activity, is also involved in controlling cell cycle progression [10,43,44]. A recent study showed that the tissue-specific upregulation of DNMT1 in pancreatic β-cells increased their proliferation ability [43]. Thus, the downregulation of DNMT1 may be associated with a decrease in the level of gDNA methylation and a decrease in the proliferation rate of INS-1E cells that are treated with the SCD1 inhibitor and palmitate, which was previously demonstrated [19]. The opposite effects of SCD1 ablation on gDNA methylation levels in β-cells and adipocytes suggest that the mode of the regulation of DNA methylation by SCD1 is specific to certain tissues and may differ between β-cells and insulin-sensitive tissues. The divergent impact of SCD1 inhibition in T2D-relevant tissues has been widely recognized. The accumulation of SFAs by SCD1 inhibition can promote inflammation and pancreatic β-cell failure [15,17,18,45]. The knockdown of SCD1 protects against diet-induced obesity and hepatic steatosis. SCD1 deficiency in skeletal muscle and adipose tissue increases the rate of fatty acids (FA) β-oxidation and glucose utilization, thereby improving insulin sensitivity and basal metabolic rate [46,47].

To date, several studies have shown that SCD1 deficiency triggers activation of the AMPK signaling pathway in the liver [48], skeletal muscles [20], and cancer cell lines [49,50]. The AMPK-mediated actions of SCD1 also include autophagy-dependent cell growth and death [49,50], FA β-oxidation [48], and histone acetylation [20]. Consistent with previous studies of other tissues, we found that the loss of SCD1 expression activated AMPK in pancreatic islets and INS-1E cells. In accordance with a study of isolated rat islets and MIN6 cells [51], we found that palmitate treatment lead to the activation of AMPK in INS-1E β-cells. Furthermore, AMPK activation enhanced SIRT1 protein expression and activity under the present experimental conditions, which is consistent with previous studies that described reciprocal interactions and the interdependence of these two metabolic sensors [20,52,53]. However, the molecular mechanism of the SCD1-dependent control of DNA methylation is still unknown. Given that the AMPK/SIRT1 axis mediates some effects of SCD1 deficiency in insulin-sensitive tissues [20,48], further characterization of the roles of AMPK and SIRT1 in the regulation of DNA methylation in SCD1-deficient β-cells is an attractive area of investigation. Palmitate can also influence the acetylation of multiple proteins in clonal β-cells [54]. We found that SIRT1 interacted with DNMT1 in pancreatic β-cells and mediated more intensive DNMT1 deacetylation in cells that were treated with the SCD1 inhibitor compared with control cells. In contrast to SCD1 inhibition, treatment with palmitate did not decrease the level of DNMT1 acetylation in INS-1E cells. Therefore, the opposing acetylation status of DNMT1 in INS-1E cells that were incubated with the SCD1 inhibitor, relative to INS-1E cells that were stimulated with palmitate, may be related to the differential regulation of DNMT1 activity and may explain more severe changes in gDNA levels in SCD1-deficient cells. However, considering the divergent effect of single lysine deacetylation on DNMT1 function and the fact that SIRT1 is not the only DNMT1 deacetylase [9,10], further research is needed to precisely verify the role of SIRT1-dependent DNMT1 deacetylation on methyltransferase activity in palmitate-treated β-cells.

**Figure 7:**
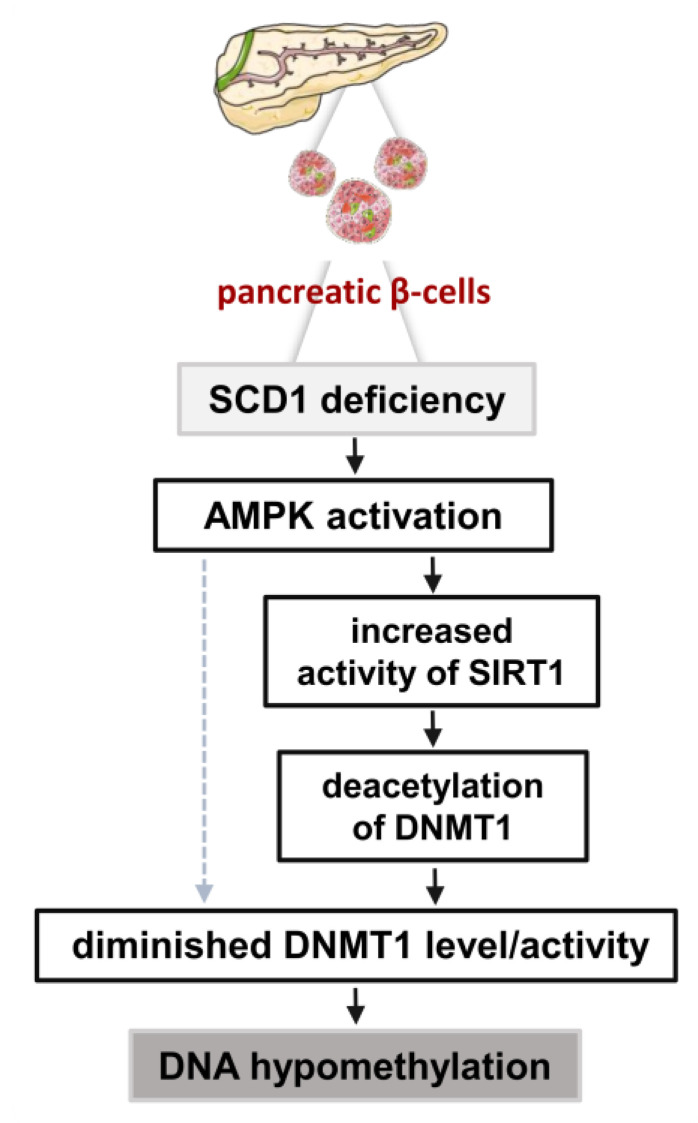
Impact of SCD1 deficiency on gDNA methylation levels in pancreatic β-cells. SCD1 downregulation in INS-1E cells results in the activation of AMPK, upregulation of SIRT1, and promotion of DNMT1 deacetylation. AMPK activation and SIRT1-dependent changes in DNMT1 acetylation status trigger the loss of DNMT1 function and stability, consequently leading to gDNA hypomethylation. The dashed arrow indicates the potential direct (independent of SIRT1) control of gDNA methylation levels by AMPK.

Lastly, we investigated whether activation of the AMPK/SIRT1 signaling cascade mediates the influence of SCD1 on DNA methylation in pancreatic β-cells. Recent studies suggest that metabolic state can directly influence DNA methylation via the ability of AMPK to phosphorylate epigenetic modifying enzymes, such as DNMT1 [55] and TET2 dioxygenase [56]. Studies of cancer cell lines showed that AMPK regulates gDNA methylation by affecting the availability of a methyl group donor that is required for DNMTs [57]. Furthermore, the AMPK-mediated inhibition of transcription factor Sp1, resulted in lower levels of DNMT1 gene transcription [58]. Interestingly, we found that AMPK inhibition resulted in a decrease in SIRT1 protein levels, an increase in DNMT1 protein levels, and an increase in gDNA methylation levels in SCD1-defcient INS-1E cells. The present results indicate that the maintenance of DNA methylation patterns in pancreatic β-cells may be regulated by SCD1 in an AMPK/SIRT1-dependent manner through the regulation of DNMT1. The proposed molecular mechanism that is involved in the SCD1-dependent control of DNA methylation in INS-1E cells, which requires the coordinated activation of AMPK and SIRT1, is presented in Figure 7.

Altogether, the present results indicate that SCD1 controls DNA methylation patterns in pancreatic islets and β-cells. Our findings uncover a novel pathway whereby SCD1 dynamically regulates DNA methylation in pancreatic islets and controls DNMT1 through the AMPK/SIRT1 axis. Our findings provide additional mechanistic insights into the role of SCD1 in maintaining DNA methylation patterns in β-cells in response to lipotoxicity and diabetes progression. The current study sheds light on the mechanisms that regulate the epigenome in pancreatic β-cells. The elucidation of such mechanisms is crucial for the design of T2D treatment approaches that can circumvent epigenetic barriers.

## 4. Materials and Methods

### 4.1. Animals and Pancreatic Islet Isolation

The generation of SCD1^−/−^ knockout mice was previously described [59]. Male wildtype (WT) C57/BL6 and SCD1^−/−^ mice on a B6 background (10 weeks of age, *n*  =  6) were fed a standard laboratory chow diet (Sniff, Germany) and water ad libitum. The mice were sacrificed by cervical dislocation, and pancreatic islets were isolated via intraductal infusion. Briefly, the pancreas was perfused by injecting 5 mL of ice-cold collagenase (Sigma, St. Louis, MO, USA) solution in Hank’s Balanced Salt Solution (HBSS) into the clamped pancreatic duct. Once inflated and removed, pancreatic tissue was incubated in 3 mL of collagenase/HBSS at 37 °C in a water bath for 10–15 min to digest exocrine tissue. Digested tissue was then washed twice with HBSS and collected in a 70 μm cell strainer. The pancreatic islets were separated from remaining tissue by density centrifugation in a histopaque-1077 (Sigma) gradient. The histopaque was then removed from the islet suspension by a series of rinses in HBSS. Islets were then handpicked using a stereomicroscope and stored at −80 °C for further analyses. All protocols used in this study were approved by the First Local Ethical Committee for Animal Experiments in Warsaw (Permit number: 37/2016, approved 21/01/2016).

### 4.2. Materials

The primary antibodies were obtained from the following suppliers: SCD1 (catalog no. 2283, Cell Signaling Technology, Hertfordshire, UK), phosphorylated AMPKα (pAMPK; catalog no. 2531, Cell Signaling Technology, Hertfordshire, UK), DNMT1 (catalog no. 5032, Cell Signaling Technology, Hertfordshire, UK), DNMT1-ChiP Grade (catalog no. ab13537, Abcam, Cambridge, UK), AMPKα1/2 (catalog no. sc-25792, Santa Cruz Biotechnology, Santa Cruz, CA, USA), acetyl-lysine (catalog no. NB100-74339, Novus Biologicals, Abingdon, UK), SIRT1 (catalog no. 07-131, Millipore, Billerica, MA, USA), and β-actin (catalog no. 3854, Sigma, St. Louis, MO, USA). Secondary peroxidase-conjugated goat anti-rabbit IgG (catalog no. 67437) and goat anti-mouse IgG (catalog no. 115-035-146) were obtained from MP Biomedicals (Irvine, CA, USA) and Jackson ImmunoReasearch Laboratories (West Grove, PA, USA), respectively. Secondary antibodies conjugated to Alexa Fluor-488 and Alexa Fluor-568 were purchased from Invitrogen (Carlsbad, CA, USA). The other chemicals were purchased from Sigma unless otherwise specified.

### 4.3. INS-1E Cell Culture and Chronic Treatments

INS-1E cells (rat insulin-secreting pancreatic β-cell line) were a generous gift from Dr. Pierre Maechler (University of Geneva, Geneva, Switzerland). The cells were cultured in complete RPMI medium supplemented with 5% heat-inactivated fetal bovine serum, 1 mM sodium pyruvate, 10 mM HEPES, 2 mM glutamine, 50 μM 2-mercaptoethanol, 100 IU/mL penicillin, and 100 μg/mL streptomycin and maintained in a 5% CO_2_ atmosphere at 37 °C. To inhibit SCD1 activity, the cells were preincubated with 2 μM of the SCD1 inhibitor A939572 (Biofine International, Blain, WA, USA) for 4 h and then co-supplemented with 0.4 mM bovine serum albumin (BSA)-conjugated palmitic acid for 16 h. As an indicator of SCD1 activity, we measured desaturation ratios of palmitate (16:0) and stearate (18:0), which are preferred substrates of SCD1, as described previously [19]. To silence SCD1 expression, INS-1E cells were grown for 24 h in antibiotic-free media and reverse-transfected with 60 ng of siRNA (given amount was used for transfection of 1 × 10^6^ cells in one well of a 6-well plate) against SCD1 (catalog no. s73339, Ambion, Houston, TX, USA) for 72 h using Lipofectamine 2000 (0.5 μL/cm^2^; Invitrogen). Silencer negative control #1 siRNA (Ambion) was applied as a negative control. The silencing efficiency was measured 72 h after transfection using real-time PCR and Western blot. Palmitate was added for the last 16 h before sample collection. To inhibit AMPK before SCD1 inhibition or SCD1 silencing, the cells were preincubated for 1 h with 10 µM compound c (Sigma) and treated with compound c throughout the entire procedure. As a vehicle control for palmitate, INS-1E cells were grown in medium that was supplemented with 7.5% BSA. Dimethyl sulfoxide (DMSO) was used as a vehicle control for the SCD1 inhibitor and compound c.

### 4.4. DNA Extraction and Global DNA Methylation Assessment

DNA was extracted using the DNeasy Blood and Tissue Kit (Qiagen, Germantown, MD, USA) according to the manufacturer’s instructions. The global DNA (gDNA) methylation profile was determined using the EpiJet Global Methylation kit (Thermo Scientific, Waltham, MA, USA). Briefly, to cleave gDNA, an isoschizomeric pair of restriction enzymes (MspI/HpaII) was used. MspI and HpaII restriction enzymes are characterized by different sensitivities to CpG methylation. When cytosine methylation within the 5′-CCGG-3′ internal CpG tetranucleotide sequence occurs, cleavage with HpaII is blocked. Otherwise, cleavage with MspI is unaffected. The cleavage results were visualized on 1% agarose gel. The effect of SCD1 inhibition on gDNA methylation levels was also assessed by Raman spectroscopy as described below.

### 4.5. Isolation of Metaphase Chromosomes

Metaphase chromosomes from INS-1E cells were obtained using classic cytogenetic methods. To arrest cells in metaphase, 5% colcemid (Max Karyo Colcemid Solution, Thermo Scientific) was added to the growth medium. After 2 h of incubation, the cells were rinsed twice with phosphate-buffered saline (PBS) and trypsinized. The collected cells were incubated with 0.75 mM KCl, fixed in a mixture of glacial acetic acid and methanol (1:3), and deposited onto CaF_2_ windows (Crystran, IR grade, Ø 20 mm). Samples were air dried before Raman measurements.

### 4.6. Raman Spectroscopic Measurements of Chromosomes and DNA Methylation

The quality of metaphases and single chromosomes were assessed before spectroscopic Raman measurements using an AFM microscope (NT-MDT) in non-contact mode (NSG01 Si probes). Based on the AFM topographical analysis of the obtained metaphases, the first pair of chromosomes was chosen from each metaphase for further Raman investigation. Single chromosome no. 1 was identified in metaphases based on their size and morphology. Spectroscopic Raman measurements of single chromosomes were performed using an integrated AFM–Raman (NT-MDT-Renishaw) system. Raman maps were obtained using an inVia Renishaw spectrometer that was equipped with an EMCCD detector (Back Illuminated, Deep Depletion CCD, Rapide 1600 × 200) with a Leica confocal microscope (100× magnification, NA = 0.85). Raman maps were collected using a green 532 nm edge laser (regular mode, maximum power = 3 mW, grating = 1800 L/mm) with a pixel size of 0.5 × 0.5 µm. Furthermore, using the same setup, droplets of gDNA solution from INS-1E cells were measured and considered as reference data. During the experiments, Raman spectra of DNA and chromosomes were analyzed in the spectral range of 3200–800 cm^−1^.

The AFM topographic images were analyzed using SPIP software (Image Metrology, Lyngby, Denmark). DNA and chromosome spectra were analyzed using WIRE 4.2 software (Renishaw). Cosmic rays were first removed based on the Nearest Neighbor algorithm, and baseline was subtracted using the Intelligent fitting algorithm. Subsequently, spectra were smoothed (Savitzky-Golay, 11 pt, 2nd order), and Empty Modeling (Empty modelling™, WiRE 4.2 software, Renishaw, UK) analysis was performed on Raman chromosome maps, which allowed us to obtain spectra of chromosomes and substrates with cellular debris. Empty modelling is a chemometric method, which enables the extraction of spectra of the key components from the Raman map. The algorithm starts from an initial estimation that the spectral values of a first component of the sample are all equal, which is an ”empty model” assumption and then resolves that component. In the next steps, the algorithm successively and iteratively resolves other components based on already resolved spectra. In our analysis, it was used to avoid the influence of cellular contamination. The resulting substrate spectrum, obtained using empty modelling, was subtracted from each spectrum of the Raman map with the appropriate coefficient by resetting the characteristic bands for the substrate. Afterward, a spatial distribution of the DNA, proteins (mainly histones), and -CH_3_ groups was calculated using OriginPro 2017 (OriginLab) and Gwyddion 2.41 software (Czech Metrology Institute). The distribution was calculated as the integral of the band characteristic for the stretching of bonds between phosphate and oxygen atoms in the DNA backbone in the spectral range of 1280–1215 cm^−1^, the band characteristic for amide I in the spectral range of 1695–1630 cm^−1^, and the band characteristic for -CH_3_ group stretching in the spectral range of 2900–2850 cm^−1^. Subsequently, OPUS 7.5 software (Bruker) was used for unit vector normalization (in the spectral range of 1750–1030 cm^−1^) of spectra that were collected from chromosomes and DNA solutions. An estimation of global methylation level in gDNA and chromosomes was performed based on the integral calculation of the band characteristic for vibrations of -CH_3_ groups in the spectral range of 2910–2870 cm^−1^ and 2900–2850 cm^−1^, respectively. Obtained results were normalized to the integral of the band characteristic for vibrations of PO_2_^−^ groups of DNA backbone in the spectral range of 1110–1030 cm^−1^ in case of DNA solutions and in the spectral range of 1110–1070 cm^−1^ in case of chromosomes.

### 4.7. INS-1E Immunostaining

INS-1E cells were grown on 0.001% poly-L-ornithine-coated coverslips in 24-well plates. The cells were washed with PBS and fixed with 4% paraformaldehyde for 15 min at room temperature. Fixed cells were then permeabilized with 0.2% Triton X-100 for 30 min, rinsed with PBS, and blocked with buffer that consisted of 3% BSA in PBS for 1 h. To obtain dual immunostaining, primary antibodies for DNMT1 (Alexa 488) and insulin (Alexa 568) were co-incubated in blocking buffer overnight. The next day, the cells were washed three times in PBS and exposed to appropriate fluorescently labeled secondary antibody for 1 h. After additional rinses with PBS, the cells were co-stained with 4′,6-diamidino-2-phenylindole (DAPI) to identify nuclei. Fluorescence images were acquired using a Zeiss spinning disk confocal microscope and then analyzed using ImageJ software.

### 4.8. Co-Immunoprecipitation Assay

INS-1E cells were homogenized in precipitation lysis buffer (20 mM Tris (pH 8.0), 75 mM NaCl, 15 mM MgCl_2_, 1 mM ethylenediaminetetraacetic acid (EDTA), 0.5% Nonidet P-40, 10% glycerol, 5 μg/mL pepstatin A, 10 μg/mL leupeptin, and 1.4 μg/mL aprotinine). Homogenates that contained 500 μg of proteins were incubated overnight at 4 °C with 20 μL of pre-washed protein A/G PLUS-agarose beads (Santa Cruz Biotechnology) and 1 μg of SIRT1 or DNMT1 (Abcam) antibodies or IgG alone (negative control samples). The immunoprecipitations were washed six times with precipitation lysis buffer, resuspended in electrophoresis sample buffer, and boiled for 10 min at 95 °C. The aliquots were then analyzed by Western blot using an antibody against acetylated lysine (AcLys).

### 4.9. Quantification of Global Acetylation of Histone H4K16

INS-1E cells (1 × 10^7^) were harvested and lysed on ice in 1 mL of TEB buffer (PBS that contained 0.5% Triton X-100, 2 mM phenylmethylsulfonyl fluoride (PMSF), and 0.02% NaN_3_). After lysis, histone core proteins were obtained from the remaining cell pellet by acidic extraction with 0.5 N HCl + 10% glycerol on ice for 30 min. Subsequently, probes were centrifuged at 12,000 rotations per minute for 5 min at 4 °C, and the supernatant fraction was collected. Histone core proteins were then precipitated from the supernatant fraction with acetone at −20 °C overnight. Extracted core histone pellets were air-dried and dissolved in high-performance liquid chromatography-grade distilled water and stored at −80 °C. The level of histone H4 acetylation at lysine 16 was colorimetrically detected using the EpiQuik Global Acetyl Histone H4K16 Quantification Kit (Epigentek, Farmingdale, NY, USA) according to the manufacturer’s instructions.

### 4.10. Gene Expression Analysis

For real-time reverse-transcription polymerase chain reaction (RT-PCR), RNA was isolated from the experimental samples using the Total RNA Mini Plus Concentrator (A&A Biotechnology, Gdynia, Poland) according to the manufacturer’s protocol. DNase-treated RNA was reverse-transcribed using the RevertAid First Strand cDNA Synthesis Kit (Thermo Scientific). To evaluate SCD1 mRNA expression levels, optimized primers that targeted the *Scd1* gene (5′-ACATTCAATCTCGGGAGAACA-3′ (forward) and 5′-CCATGCAGTCGATGAAGAAC-3′ (reverse)) were used. Real-time quantitative PCR was performed using the RT-PCR 7900 HT Real Time PCR System, and SYBR green was used for detection. The relative expression of each sample was determined after normalization to GAPDH using the ΔΔCt method.

### 4.11. Western Blot Analysis

The experimental samples were collected and lysed for 30 min in ice-cold buffer (50 mM Tris-HCl, 5 mM EDTA, 1% Triton X-100, 10 mM sodium fluoride, and 150 mM NaCl) that contained inhibitors of proteases and phosphatases (10 μg/μL leupeptin, 5 μg/μL pepstatin A, 2 μg/μL aprotinine, 1 mM sodium orthovanadate, and 1 mM PMSF). The protein content in the lysates was determined using a protein assay (Bio-Rad, Hercules, CA, USA) with BSA as the reference. The prepared samples were loaded onto 10% sodium dodecyl sulfate-polyacrylamide gel electrophoresis gels. The separated proteins were then transferred to PVDF membranes (Millipore, Billerica, MA, USA) and blotted using appropriate antibodies. Proteins were visualized using SuperSignal West Pico PLUS Chemiluminescent Substrate (Thermo Scientific) and quantified by densitometry.

### 4.12. Statistical Analysis

Statistical significance was assessed using GraphPad Prism 8.3.0 software. Multiple comparisons were performed using one-way analysis of variance (ANOVA) followed by Tukey’s post hoc test. Unpaired *t*-tests were used when two groups were compared. Values of *p* < 0.05 were considered statistically significant. The data are expressed as mean ± SD.

## Abbreviations

AFM	Atomic force microscopy
AMPK	AMP-activated protein kinase
DNMT1	DNA methyltransferase 1
FA	Fatty acids
MUFA	Monounsaturated fatty acids
SAM	S-adenosyl methionine
SCD1	Stearoyl-CoA desaturase 1
SFA	Saturated fatty acids
SIRT1	NAD-dependent deacetylase sirtuin-1
T2D	Type 2 diabetes mellitus
TET	Ten-eleven translocation dioxygenase
